# Microemulsion-based topical hydrogels containing lemongrass leaf essential oil (*Cymbopogon citratus* (DC.) Stapf) and mango seed kernel extract (*Mangifera indica* Linn) for acne treatment: Preparation and in-vitro evaluations

**DOI:** 10.1371/journal.pone.0312841

**Published:** 2024-10-31

**Authors:** Ngoc Nha Thao Nguyen, Thi Trang Dai Nguyen, Duc Linh Vo, Dang Tuyet Minh Than, Gia Phuoc Tien, Duy Toan Pham

**Affiliations:** 1 Faculty of Pharmacy, Can Tho University of Medicine and Pharmacy, Can Tho, Vietnam; 2 DHG Pharmaceutical Joint-Stock Company, Can Tho, Vietnam; 3 Hycha Vietnam Limited Company, Can Tho, Vietnam; 4 Department of Health Sciences, College of Natural Sciences, Can Tho University, Can Tho, Vietnam; Public Library of Science, UNITED KINGDOM OF GREAT BRITAIN AND NORTHERN IRELAND

## Abstract

Current treatments for severe acne include combinations of synthetic anti-inflammatory and antibacterial drugs, which possess numerous side effects. Therefore, this study developed microemulsion-based hydrogel containing lemongrass leaf essential oil (*Cymbopogon citratus* (DC.) Stapf) and mango seed kernel extract (*Mangifera indica* Linn) as a potential natural therapy for inflammatory acne. To this end, the microemulsions were first prepared using pseudo-ternary phase diagrams with soybean oil and coconut oil, cremophor RH40, and PEG 400. The optimal formula could load 1% lemongrass oil and 10% mango extract, possessed a spherical droplet size of ~18.98 nm, a zeta potential of -5.56 mV, and a thermodynamic stability. Secondly, the microemulsion-based hydrogel was developed by simple mixing the optimal microemulsion in carbopol-940 hydrogel (3.5% w/w). The product showed a viscosity of ~3728 cPs, a pH of 5.4-6.2, a spreadability of ~24 cm, an in-vitro Franz-cell cumulative release rate of ~80% for polyphenol content and ~60% for citral within 12 h, and a good physicochemical stability of > 3 months. Thirdly, the skin compatibility/irritability of the microemulsion-based hydrogel was determined by the HET-CAM assay, which showed non-irritation level. Finally, the anti-inflammatory activities of the hydrogel, using heat-induced BSA denaturation assay and LPS-stimulated RAW 264.7 NO inhibition assay, was 4-times higher than that of the reference drug Klenzit-C^®^ (adapalene and clindamycin gel). Moreover, the hydrogel possessed strong anti-biofilm activity in *Cutibacterium acnes*, comparable with Klenzit-C^®^. Conclusively, the microemulsion-based hydrogel containing lemongrass oil and mango seed extract demonstrated much potentials to be a promising natural drug for acne treatment.

## Introduction

Acne vulgaris, a common skin inflammatory disorder affecting approximately 9.4% of the global population [1], has caused significant impacts on patient mental health and self-confidence [2]. Two main causes underlying the onset of acne include hormonal fluctuations (i.e., puberty period) and bacterial infections, generally by the *Cutibacterium acnes* (previously known as *Propionibacterium acnes*) [3]. The latter cases could initiate the severe form of acne, resulted in inflamed blackheads and whiteheads, seborrhoea, large cysts, and nodules, which causes swelling, scarring, and pain [4]. Treatments for acne consist of the combinations of antibiotics to eradicate *Cutibacterium acnes* (i.e., benzoyl peroxide, clindamycin, tetracycline) and anti-inflammatory agents to alleviate the inflammation symptoms (i.e., tretinoin, adapalene, tazarotene), in the forms of topical and/or oral administrations, dependent on the disease stages [5,6]. Among them, the current (as of 2023) first-line treatment for severe acne and recommended treatments for moderate acne is isotretinoin, a derivative of vitamin A that affects almost all acne developmental stages, namely the comedon growth, the cell differentiation and apoptosis, and the bacterial infections [2,5]. Nevertheless, these acne therapies have various side effects such as dry lips, xerosis, xerostomia, photosensitivity, diarrhea, dizziness, nausea and vomiting, and rash, among others [7,8]. Moreover, the conventional topical products for acne (i.e., gel, cream, and ointment) mostly possess limited efficacy due to inadequate drug permeation through the skin, and skin irritation due to the uses of many excipients [9,10]. Thus, to overcome these issues, on the one hand, natural products (i.e., herbal extracts) are employed to substitute the synthetic anti-acne drugs, which potentially reduce the drug side effects; on the other hand, microemulsion-based hydrogel could be utilized to circumvent the conventional topical dosage forms disadvantages of low permeability and high irritability.

Regarding the natural products, numerous herbal/traditional medicine have been used for the biomedical purposes [11,12]. Among them, lemongrass oil (*Cymbopogon citratus* (DC.) Stapf) and mango seed extract (*Mangifera indica* Linn), the commonly found plants in the Southeast Asia region, demonstrate interesting anti-inflammatory and antibacterial actions that would benefit acne treatments. Lemongrass leaves contain a good source of various bioactive compounds, including alkaloids, terpenoids, flavonoids, phenols, saponins, and tannins [13,14], which possess numerous therapeutic actions including antiviral [15], antimicrobial [14,16], antioxidant [17], and anti-inflammatory [18]. Specifically, for the acne treatments, lemongrass oils, citral compounds (geranial and neral), provide high efficacy against acne-causing bacteria *Cutibacterium acnes* and *Staphylococcus epidermidis* with a minimum inhibitory concentration (MIC) of 0.125-0.250 mg/mL [19]. Citral also shows anti-inflammatory actions by inhibiting the productions of the biomarkers vascular cell adhesion molecule 1 (VCAM-1), interferon gamma-induced protein 10 (IP-10), interferon-inducible T-cell alpha chemoattractant (I-TAC), and monokine induced by gamma interferon (MIG) in both human and animal cells [18]. Similarly, mango seed kernel extract, mainly composed of carotenoids, tocopherol, polyphenols (mangiferin, hesperidin, rutin, quercetin, and kaempferol), and phenolic acids (gallic acid, caffeic acid, and ellagic acid), possesses high antioxidant, anticancer, and antimicrobial properties [20,21]. In terms of acne treatments, the mango seed ethanolic fractions exhibited strong antimicrobial effect against *Cutibacterium acnes* with an MIC and minimum bactericidal concentration (MBC) of 1.56 mg/mL and 12.50 mg/mL, respectively [22]. Moreover, the extract yielded significant anti-inflammatory effects on LPS-induced RAW 264.7 cells [23]. Conclusively, these two natural products, the lemongrass essential oil and the mango seed kernel extract, were selected in this study to be loaded into the microemulsion-based hydrogel as an alternative acne treatment.

Regarding the formulation issue, micro-/nano-based formulas have been extensively studied in biomedical areas [24–26]. Among them, the utilizations of microemulsion-based topical hydrogels have gained increasing attentions due to their versatility, biocompatibility, high permeability, and lipophilic-drug encapsulability [27,28]. Microemulsions are thermodynamically stable nano-micelles consisting of water, oil, surfactants, and co-surfactants, which are suitable for topical drug delivery to manage a variety of skin disorders such as acne [2,4,27,29,30]. Although microemulsions are well-known for their potentials to increase the permeation of drugs through the skin, most studies focus on the encapsulation and delivery of the pure drugs. To the best of our knowledge, no study has been investigated the microemulsion ability to load and co-deliver natural extracts for acne treatment.

Ultimately, to fill in the knowledge gap of co-delivery natural products using microemulsion and to explore the system potentials as a novel topical anti-acne pharmaceutical, this study developed and characterized microemulsion-based topical hydrogels containing lemongrass leaf essential oil and mango seed kernel extract. The extracts were prepared using simple distillation/maceration methods. Then, the extract loaded microemulsions were prepared using pseudo-ternary phase diagrams titration technique, and characterized in terms of droplet size, shape, zeta potential, and thermodynamic stability. Next, the microemulsion-based hydrogel was developed by the mixing method, and determined the viscosity, pH, spreadability, in-vitro Franz-cell release rate, and physicochemical stability. Finally, the skin compatibility/irritability, anti-inflammatory activities, and anti-biofilm formation actions of the product were evaluated.

## Materials and methods

### Materials

Fresh seeds of mango ripened fruits and lemongrass leaves (6 months old) were collected in O Mon, Can Tho, Vietnam, from March to April, 2021. Both herbs were identified and authenticated at the Department of Pharmacognosy-Botany-Traditional medicine, Can Tho University of Medicine and Pharmacy, where the voucher specimen were preserved. The mango seeds were washed, dried at 60°C (humidity of 8.3%), finely milled, and kept at room temperature until use. The lemongrass leaves were washed, cut into small pieces, dried, and kept at room temperature until use.

Standard mangiferin and diclofenac were purchased from Vietnam National Institute of Drug Quality Control. Standard citral (geranial and neral) and gallic acid (purity > 99%) were acquired from Sigma-Aldrich (Singapore). Crodamol PC (propylene glycol dicaprylocaprate) was bought from Croda (Spain). Tween 80 (polysorbate 80), span 80, and poly(ethylene glycol) 400 (PEG 400) were purchased from B.L. Hua & Co. Ltd. (Thailand). Cremophor RH 40 (PEG-40 hydrogenated castor oil) was imported from BASF (Germany). Soybean oil, coconut oil, castor oil, caprylic acid, and carbopol 940 were bought from 3C Cosmetic (Vietnam). Levofloxacin, *λ*-carrageenan, and Folin-Ciocalteu reagents were purchased from Sigma-Aldrich Chemical Co. (USA). All other chemicals and solvents used were of reagent grades or higher.

### Mango seed kernel extraction and chemical quantitation

#### Mango seed kernel extraction

From 3.5 kg of the dried mango seed kernels, after the grinding and milling process, 3 kg of the mango seed kernel powder was obtained and macerated in 24 L ethanol 96% (v/v) at room temperature for 24 h. The obtained filtrates were condensed under vacuum in a rotavapor (Heidolph, USA) at 40°C to get 159.6 g extract. The extract was stabilized under vacuum drying cabinet and stored at 4°C for further experiments.

#### Total phenolic content quantitation

The total phenolic content of the extract was determined by UV-Vis spectroscopy at a wavelength of 760 nm after reaction with Folin-Ciocalteu reagent, following standard procedure [31]. Briefly, 5 mL of Folin-Ciocalteu reagent (10% w/v) was added to 1 mL of the mango seed extract, incubated in the dark for 5 min at room temperature, followed by the addition of 4 mL of Na_2_CO_3_ solution (7.5% w/v) and another 1-h incubation. Finally, the mixture absorbance was UV-Vis spectroscopic measured and the total polyphenol content in the extract was calculated based on the gallic acid standard curve (y = 0.0102x - 0.0056, R^2^ = 0.9984). The results were expressed as mg gallic acid equivalents (GAE) per g extract.

### Lemongrass leaf essential oil extraction and chemical determination

#### Lemongrass leaf essential oil extraction

The lemongrass leaf essential oil was extracted by the direct distillation with water [13]. The extraction was performed with 5 kg of dried leaves powder for 4 h at 100°C. The obtained lemongrass oil was then kept in the dark at room temperature until uses.

#### Essential oil chemical quantitation

To determine and quantify the essential oil main compositions, gas chromatography-mass spectrometry (GC-MS/MS) technique was employed [32], using the machine model QP2010 (Shimadzu, Japan), with a DB-5MS column (30 m × 0.25 mm), an injection temperature of 280°C, an ionization temperature of 250°C, a carrier gas of helium, a flow rate of 1 mL/min, and a sample volume of 1 μL. The temperature program was set at gradient mode (60°C for the first 2.5 min, increased to 180°C (10°C/min), kept at 180°C for 5 min, increased to 280°C (20°C/min), and kept at 280°C for 1 min). The MS detector was set at an ionization temperature of 280°C, with time-of-flight mode, and the recorded signal in the range of 15-500 m/z.

### Microemulsion preparation

Firstly, to find the most suitable microemulsion compositions, the solubility of lemongrass oil in different oil phases (soybean oil, castor oil, coconut oil, isopropyl myristate, and caprylic acid), surfactants (cremophor RH40, span 80, and tween 80), and co-surfactants (PEG 400 and propylene glycol), was determined using the shake-flask method [33]. For this, excess amount of the lemongrass oil was mixed with 1 g of each excipient and stirred at 37 ± 0.5°C for 24 h. Excipients that could dissolve the highest amount of lemongrass oil (i.e., forming the homogeneous mixtures with the highest transmittances) were chosen for preparing the microemulsions.

Secondly, the pseudo-ternary phase diagrams were created, using the Chemix School 7.0 software, by the water titration method at room temperature under controlled stirring [2]. Mixtures of the optimal surfactants/co-surfactants (S_mix_) and the optimal oil phase was homogenized at different ratios (9:1, 8:2, 7:3, 6:4, 5:5, 4:6, 3:7, 2:8, and 1:9 w/w). The addition of water into the mixtures spontaneously generate turbid dispersions, which were visually assessed and determined as microemulsions, emulsions, or gels. The areas yielded the formations of clear microemulsions were selected and yellow colored in the phase diagrams.

Finally, using the constructed phase diagrams, the complete microemulsions containing lemongrass oil and mango seed extract were formulated. To this end, the lemongrass oil (0.5%, 1%, or 1.5% v/w) was dissolved in the optimal oil phase, followed by the addition of the optimal S_mix_ and adequate water amount. Then, the mango seed extract (5%, 10%, or 15% w/w) was mixed with the obtained microemulsions under magnetic stirring for 15 min to form the final product.

### Microemulsion characterizations

The microemulsions were characterized in terms of droplet size and polydispersity index (PI), zeta potential, shape, and thermodynamic stability.

#### Droplet size, PI, and zeta potential

For the droplet size and PI, the dynamic light scattering technique (DLS) was employed using the Zetasizer machine (Nano ZS, Malvern, UK) at a laser wavelength of 633 nm [34]. The average size was calculated by the machine incorporated software, using the Stokes-Einstein equation. For the zeta potential, the phase-analysis light scattering method was utilized. The system electrophoretic mobility was measured based on the phase changes of the interferences between light scattered by the sample and the reference beam, and converted to zeta potential by Smoluchowski equation. The microemulsions samples were diluted in water until a count rate of approximately 200 kilo counts per second (kcps). Then, the measurements were conducted following the machine operational procedures.

#### Droplet shape

For the droplet shape, transmission electron microscopy (TEM) technique was used (Tecnai G TF20, 200 kV, Philips, USA) [34]. The microemulsion (20 μL) was dropped onto the carbon-coated copper grid, negatively stained with 10 μL of uranyl acetate (2%, w/v), air-dried, and observed under TEM nitrogenic atmosphere.

#### Thermodynamic stability

Regarding the microemulsion thermodynamic stability, the system was centrifuged (3000 rpm, 15 min) and visually observed for phase separation and precipitation (if any) [33]. Additionally, the microemulsion was also subjected to six heating-cooling cycles (at 4°C and 45°C, respectively), 48 h/cycle, and the sample was then centrifuged (3000 rpm for 15 min) for observations.

### Microemulsion-based hydrogel preparation

The final product, microemulsion-based hydrogel containing lemongrass oil and mango seed extract, was prepared by the simple mixing method of the optimal microemulsion and the hydrogel base [35,36]. For this, the hydrogel base (30 g) was firstly fabricated using the gelling agent carbopol 940 (5%, 6%, or 7% w/w, corresponding to 1.5 g, 1.8 g, and 2.1 g), preservative agent nipagin M (0.02 g), humectant glycerin (3.0 g), and water (aq. 30 g). The hydrogel with suitable viscosity and transparency was then used to mix with 30 g of the microemulsion. The obtained microemulsion-based hydrogel, with a final carbopol concentration of 2.5%, 3%, or 3.5% w/w), was stored at 4-8°C for further experiments.

### Microemulsion-based hydrogel characterizations

The final hydrogel was characterized in terms of appearance, viscosity, pH, spreadability, in-vitro Franz-cell release rate, and physicochemical stability.

#### Visual appearance, viscosity, pH, and spreadability

The system appearance was visually observed and recorded its color, turbidity, and transparency. The hydrogel transparency was evaluated based on the standard four-level ranking scale of (+) opaque, (++) translucent, (+++) transparent, and (++++) water-clear [37]. The hydrogel viscosity was determined at 25 ± 1°C by the viscometer Brookfield DV-E with a spindle-type apparatus (S64) at a speed of 50 rpm. The pH was checked by the pH meter (Systronics, India) with calibrated parameters using buffers at pH 4, 7, and 10. The hydrogel spreadability was determined by placing 1 g hydrogel in an area of 1 cm^2^ on a glass slide. Another slide was set on top of the previous slide, and a weight of 125 g was applied on the upper glass slide for 1 min. The hydrogel spreading diameter was then recorded [35].

#### In-vitro Franz-cell release rate

The in-vitro release profile of the hydrogel was determined using the Franz diffusion cells [38]. To this end, 0.5 g of the hydrogel (containing 5 μL of lemongrass oil and 50 mg of mango seed extract) was subjected to the donor chamber and 15 mL of phosphate buffer pH 7.4 was filled in the receptor chamber, continuously stirred, and maintained at 32 ± 0.5°C for 24 h. A cellulose acetate membrane (pore size of 0.45 μm, area of 3.14 cm^2^) was placed between the two compartments. At each time interval of 1, 2, 6, 12, and 24 h, 1 mL of the receptor solution was withdrawn, buffer refilled, and the samples were analyzed for polyphenol amount using UV-Vis spectroscopy and for citral amount using GC-MS/MS, followed the procedure described in the respective sections. The cumulative drug release amounts (%) were calculated and presented as a function of time.

#### Physicochemical stability

In terms of the physicochemical stability, the hydrogel was kept at room temperature for 3 months. At each time point of 0, 1, 2, and 3 months, the hydrogel was re-determined the appearance and drug content (polyphenol, measured by UV-Vis spectroscopy, and citral, measured by GC-MS/MS, as described previously). Any parameters that possessed a change of > 10% compared to the initial values were reported [39].

### Irritation test

To test the product skin compatibility/irritability, the HET-CAM assay was utilized, following the standard protocol [40]. Briefly, nine fertilized chicken eggs were selected (3 for the positive control (sodium dodecyl sulfate 1% w/v), 3 for the negative control (NaCl 0.9% w/v), and 3 for the hydrogel sample), incubated at 37 ± 0.5°C and 65 ± 1% relative humidity for 8 days. Then, the CAM was exposed by cutting the eggshell and removing the inner membrane. Next, the samples (200 μL) were applied on the CAM surfaces and the irritation effects were visually observed in terms of the blood vessels changes (i.e., coagulation (intra- and extra-vascular protein denaturation), vascular lysis (blood vessel disintegration), and hemorrhage (bleeding from the vessels)) at each time interval of 0 s, 30 s, 120 s, and 300 s. The sample irritation scores were determined based on **Table 1**, and the irritability level was assessed as (1) none (score of < 1), (2) slight (score of < 5), (3) moderate (score of < 9), and (4) severe (score from 9 to 21).

**Table 1:** Irritation scores of the hen’s egg-chorioallantoic membrane (HET-CAM) test.

Effect	Score		
	30 s (0.5 min)	120 s (2 min)	300 s (5 min)
Vascular lysis	5	3	1
Hemorrhage	7	5	3
Coagulation	9	7	5

### Cell culture and cytotoxicity test

Prior to the anti-inflammatory test on the LPS-induced RAW 264.7 cells, the hydrogel cytotoxicity on the cells was determined. For this, the murine macrophages RAW 264.7 cells (ATCC-TIB-71) were cultured in a high glucose Dulbecco’s Modified Eagle’s medium (DMEM, Gibco Fisher Scientific, USA) supplemented with 10% fetal bovine serum and 1% antibiotic, at 37°C and 5% CO_2_ environment. Confluent cells were sub-cultured every 3-5 days.

The cell viability was determined using the 3-(4,5-dimethylthiazol-2-yl)-2,5-diphenyl tetrazolium bromide (MTT) assay [41]. Briefly, RAW 264.7 cells were seeded into 96-well plates at a numerical concentration of 30,000 cells/well and incubated for 24 h. Then, 200 μL of the hydrogel, at different concentrations of 16, 32, 64, 128, 256, and 512 μg/mL (prepared by serial dilution technique), was added to the wells and incubated for another 4 h, followed by cell washing in phosphate buffer saline (PBS, 100 μL/well). Next, the MTT solution (5 mg/mL in PBS) was subjected to the wells (20 μL/well) and incubated for 4 h. Finally, the formed formazan crystals were dissolved by dimethylsulfoxide (100 μL/well) and the absorbance was UV-Vis spectroscopic measured at 540 nm (Varioskan, Thermofisher, Waltham, USA). The blank was the mixture of PBS and MTT without cells and the positive control was the untreated cells (i.e., 100% viability). The cell viability percentages were calculated using Eq (1).


$$\%\;Viability=\frac{Absorbance\;sample-Absorbance\;blank}{Absorbance\;positive\;control-Absorbance\;blank}x100\%$$ (1)

### In-vitro anti-inflammatory tests

To evaluate the in-vitro anti-inflammatory activities of the hydrogel, two assays were employed, including the LPS-induced RAW 264.7 cell NO inhibitory assay and the heat-induced BSA denaturation assay. The test samples included (1) the blank hydrogel (hydrogel with no extract/oil, prepared in a similar method described in **section Microemulsion-based hydrogel preparation**), (2) the hydrogel containing mango seed kernel extract and lemongrass essential oil, and (3) the commercial product Klenzit-C^®^ (adapalene and clindamycin gel).

For the NO inhibitory assay, the RAW 264.7 cells were seeded in a 96-well plate at a density of 20,000 cells/well and incubated overnight at 37°C, 5% CO_2_, and 95% humidity. Then, the cells were immersed in serum-free medium for 3 h, followed by the addition of the samples at different concentrations for 2 h, and stimulated with LPS (1 μg/mL) for another 24 h. Next, the cell culture supernatants were collected and mixed with Griess reagent (sulfanilamide 1%, N-1-naphthylenediamine dihydrochloride 0.1%) and phosphoric acid 2.5% at room temperature for 10 min. Finally, the samples absorbance was measured by UV-Vis spectroscopy at a wavelength of 540 nm using a microplate reader. The negative controls were cells with only culture medium, the positive controls were LPS-induced cells without treatments, and the reference control was cells treated with NG-methyl-L-arginine acetate (L-NMMA). The nitrite content of the samples was determined using the standard NaNO_2_ calibration curve and the NO inhibitory percentages were calculated by Eq (2). The IC50 values (concentration that inhibits 50% of NO formation) were determined using the Excel software.


$$\%Inhibitory=\frac{Absorbance\;positive\;control-Absorbance\;sample}{Absorbance\;positive\;control-Absorbance\;negative\;control}x100\%$$ (2)

Regarding the BSA assay, 150 μL of the test samples or the reference drug diclofenac were mixed with 150 μL BSA (1% w/v in PBS) and incubated at 27°C for 20 min and then at 80°C for another 20 min. The mixture absorbance was measured at 660 nm using UV-Vis spectroscopy. The control was the untreated BSA solution, which represents 100% protein denaturation. The percentage inhibition of BSA denaturation was calculated using Eq (3).


$$\%Inhibitory=\frac{Absorbance\;control-Absorbance\;sample}{Absorbance\;control}x100\%$$ (3)

### In-vitro anti-biofilm formation test

The anti-biofilm formation assay was undertaken using a human plasma protein-coated microtiter plate method, as previously described [42]. The wells were pre-coated with human plasma (diluted in carbonate buffer pH 9.6, 20% w/v) at 4°C for 24 h. Then, the plasma was removed and the pre-cultured *Cutibacterium acnes* (ATCC 6919) in tryptic soy broth supplemented with dextrose 0.5% w/v and NaCl 3.0% w/v, as well as the test samples (the blank hydrogel, the extract loaded hydrogel, and the reference product Klenzit-C^®^), were added to the wells (200 μL/well). After another incubation at 37°C for 72 h, the wells were washed twice with PBS (200 μL/well) to remove the non-adherent cells, and the adherent biofilms were fixed with 200 μL of 100% ethanol prior to staining with 100 μL of crystal violet solution (0.2% w/v in ethanol 12% v/v) for 5 min. The stain was then aspirated, and the wells were washed thrice with PBS, followed by an overnight drying step. Finally, the crystal violet was eluted with 200 μL of 100% ethanol for 10 min, and the total of 50 μL of the eluates was transferred to a new sterile polystyrene microtiter plate, followed by the UV-Vis spectroscopic measurements at 590 nm. The percentage of biofilm reduction was calculated by Eq (4), with the untreated cells were set as the control.


$$\%Biofilm\;reduction=\frac{Absorbance\;control-Absorbance\;sample}{Absorbance\;control}x100\%$$ (4)

### Statistical evaluations

All measurements were done in triplicates and the results were stated as mean ± standard deviation (SD). Differences between data were analyzed using the Student’s t-test and One-way analysis of variance (ANOVA). A p-value of < 0.05 was considered significant.

## Results and discussions

### Plant extractions and chemical quantitation

Prior to the encapsulations in the microemulsion system, the mango seed kernel polyphenols and lemongrass essential oils were extracted using the standard techniques of maceration and distillation, respectively. The mango seed extract was sticky, dark brown, and possessed a characterized odor, with an extraction yield of 8.80 ± 0.41% (w/w) and a total polyphenol content of 111.3 ± 14.2 mg GAE/g extract. These results were in accordance with the previous study that stated the mango seed extraction yields and polyphenol contents varied from 2.53 to 11.9% and from 73.8 to 399.8 mg GAE/g extract, dependent on the mango variety [43].

The lemongrass distillation process had a high extraction efficiency of 0.64 ± 0.24% (v/w), and the essential oils, analyzed by GC-MS/MS, contained mainly citral (67.68%), dodecanoic acid (4.78%), trans-geraniol (2.70%), and phellandral (1.30%). The full list of 26 compounds is presented in **S1 Fig**. Compared to the published reports, these compounds were also presented in lemongrass from other regions. However, the compound amounts were significantly different between our data (Vietnamese lemongrass) and the others (i.e., Chinese lemongrass) [13,14,16], indicating the influences of geographical and cultivation conditions on the plant bioactive components.

In summary, both the extracts were suitable for the next step, the preparations of microemulsion containing mango seed kernel extract and lemongrass essential oil. Additionally, the total polyphenol content and citral amount were selected as quantitative markers for the mango seed extract and the lemongrass oil, respectively.

### Microemulsion preparation and characterizations

From different oil phases (soybean oil, castor oil, coconut oil, isopropyl myristate, and caprylic acid), surfactants (cremophor RH40, span 80, and tween 80), and co-surfactants (PEG 400 and propylene glycol), the lemongrass oil possessed the highest solubility in soybean oil, coconut oil, cremophor RH 40, tween 80, span 80, and PEG 400, with the highest light transmittances. These data were different than results from previous study, which indicated that the lemongrass oil had a poor solubility in the soybean oil [44]. This fact suggests that the plant compositions significantly affect its solubility.

**Fig 1:**
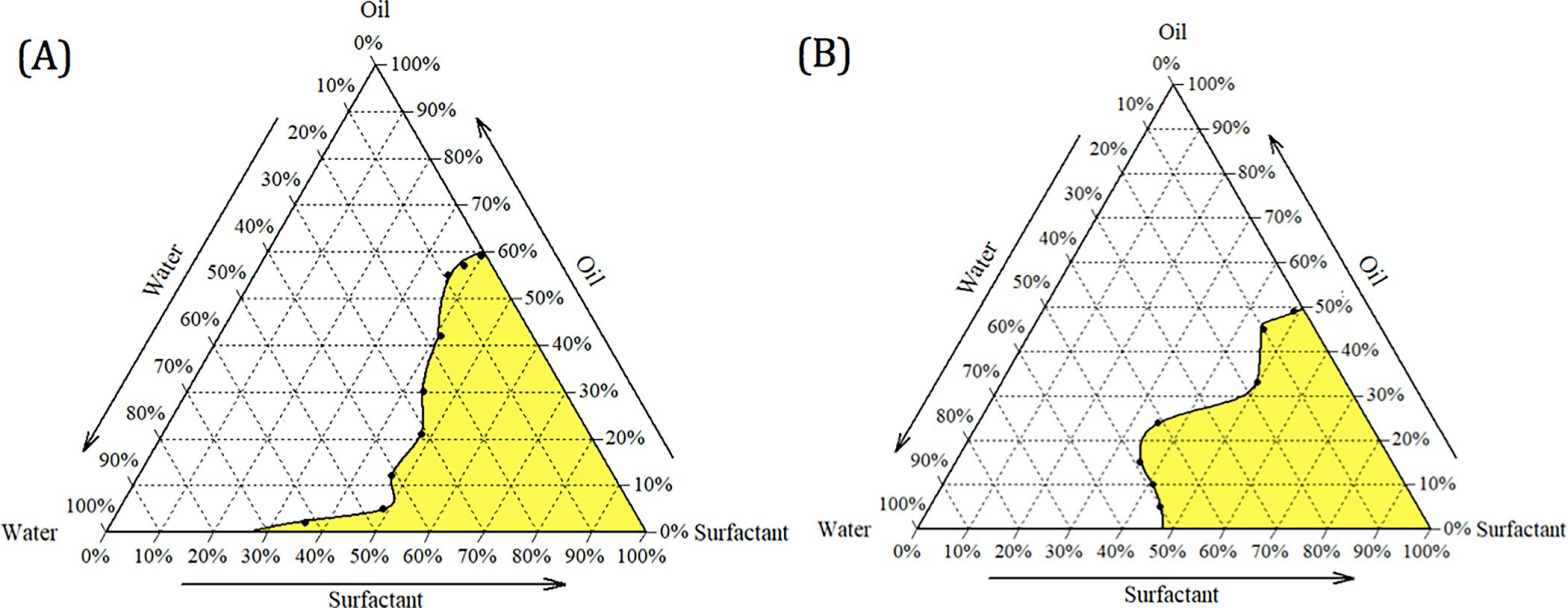
Pseudo-ternary phase diagrams of the microemulsion composed of (A) soybean oil and coconut oil (1:1) – cremophor RH40 and PEG 400 (3:1) – water, and (B) soybean oil and coconut oil (1:1) – tween 80, span 80, and PEG 400 (3:1:1) – water.

Then, mixtures of the optimal ingredients, with varying percentage ratios, were prepared at different S_mix_:oil (soybean oil:coconut oil 1:1 v/v) ratios of 9:1, 8:2, 7:3, 6:4, 5:5, 4:6, 3:7, 2:8, and 1:9 w/w, followed by the pseudo-ternary phase diagram construction by water titration method. To this end, the S_mix_:oil ratio of 1:3 w/w provided a wide microemulsion region (i.e., the area that produces stable self-emulsifying microemulsion), for both the S_mix_ of cremophor RH40 and PEG 400 (3:1 w/w), and tween 80, span 80, and PEG 400 (3:1:1 w/w/w) (**Fig 1**). To avoid the usage of excessive chemical, the S_mix_ of cremophor RH40 and PEG 400 was selected.

**Fig 2:**
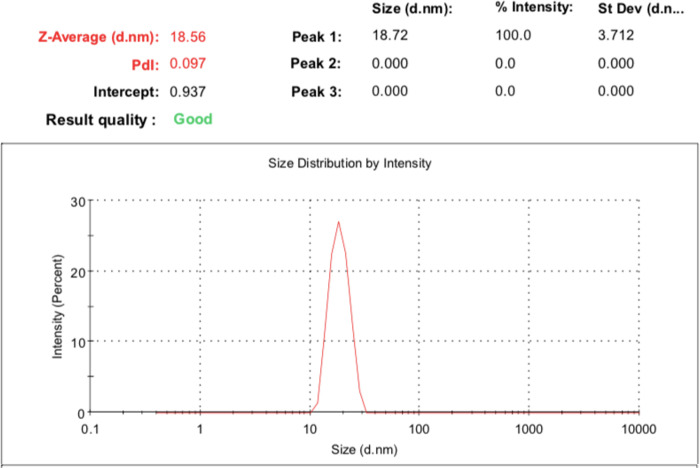
Droplet size and size distribution of the microemulsion containing mango seed kernel extract and lemongrass essential oil.

**Fig 3:**
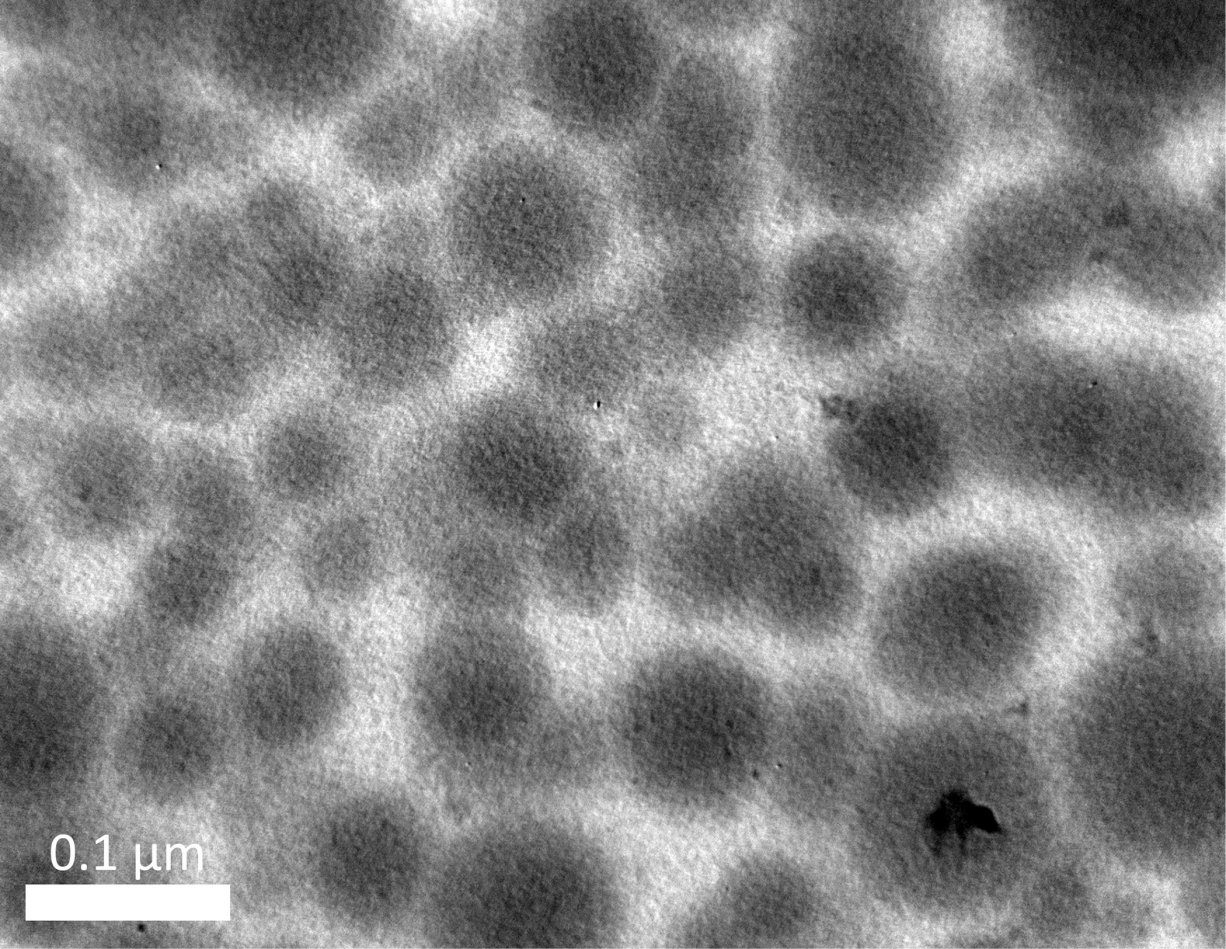
TEM image of the microemulsion containing mango seed kernel extract and lemongrass essential oil. Scale bar: 0.1 μm.

Physicochemically, the optimal formula could load up to 1% lemongrass oil and 10% mango extract, as higher loading percentages yielded phase separation and precipitation. The microemulsion possessed an average droplet size of 18.98 ± 0.62 nm, a narrow size distribution (PI = 0.095 ± 0.002, < 0.1) (**Fig 2**), and a zeta potential of -5.56 ± 0.11 mV. The small diameter of formulation could be attributed to the effect of co-surfactant molecules that lower the fluidity and surface tension of the interfacial film, thereby decreasing the radius of the nanodroplets [45,46]. Moreover, the TEM micrograph demonstrated that the droplets were spherical, homogeneous, and non-aggregated, with sizes comparable to the hydrodynamic diameters measured by the DLS method (**Fig 3**). Thermodynamically, the microemulsion remained unchanged, in terms of appearance (i.e., no phase separation and/or precipitation), transparency, and droplets sizes, after six heating-cooling cycles, indicating its high stability. Although the system zeta potential was near zero (-5.56 ± 0.11 mV), which is the favorable condition for particle aggregation and/or phase separation due to the lack of repulsive electrostatic forces [47], the present microemulsion could maintain its stability, possibly by the steric hindrance and stabilization effects of the polymer PEG 400 [48,49].

### Microemulsion-based hydrogel preparation and characterizations

Firstly, the hydrogel base was prepared at different concentrations (5%, 6%, and 7% w/w) of carbopol 940, the gelling agent. For this, all three hydrogel-base samples were homogeneous, clear, transparent (level (+++) in transparency ranking scale), and had their viscosities increased proportionally with the carbopol concentrations, from 4370 ± 215 cPs at 5%, 6769 ± 301 cPs at 6%, to 8326 ± 322 cPs at 7%. Since the hydrogel was then mixed with the microemulsion at the ratio of 1:1 w/w, the hydrogel base with a carbopol concentration of 7% was selected due to the high viscosity (8326 ± 322 cPs), appropriate gel appearances (homogeneous, clear, and transparent), suitable pH of 5.8-6.4, and acceptable spreadability of 17 ± 1 cm.

Then, the optimal microemulsion was incorporated into the hydrogel base (ratio 1:1 w/w) by the physical mixing method. The final product possessed a carbopol concentration of 3.5% w/w, a viscosity of 3728 ± 237 cPs, and a pH of 5.4-6.2, which was suitable for the skin application (the skin pH is acidic, normally at 5.12 ± 0.56) [50]. Regarding the visual appearance, the microemulsion-based hydrogel demonstrated identical properties compared to the hydrogel base (homogeneous, clear, and transparent (+++) under the normal condition), with light yellowish color of the mango extract. These results were in agreement with previous studies, which suggested that the microemulsion was successfully incorporated in the hydrogel with limited alterations to its inherent properties [33,36,51]. Moreover, the hydrogel spreadability was 24 ± 2 cm, higher than that of the microemulsion-based hydrogels in the previous studies (7.2 ± 0.01 cm [33] and 5.7 ± 0.01 cm [28]) and of the reference drug Klenzit-C^®^ (4 ± 1 cm), which could be due to the effects of the microemulsion small droplets sizes that effectively encapsulate the oil phase inside the vesicles, thus making the system more hydrophilic [52]. Furthermore, the microemulsion-based hydrogel showed higher spreadability values than those of the hydrogel base with no microemulsion (24 ± 2 cm vs. 17 ± 1 cm), possibly because of the low viscosity of the former, since a highly viscous gel could hardly spread. Overall, the hydrogel appearances, pH, viscosity, and spreadability values were appropriate for the topical applications [53,54], and could be beneficial for the acne treatment. Last but not least, the hydrogel was physicochemically stable at room temperature for at least 3 months. After 3-month storage, the hydrogel parameters of appearances and drug contents, were in the range of ± 10% compared to the initial values (**Table 2**).

**Table 2:** Stability data (3 months storage at room temperature) of the microemulsion-based hydrogel containing mango seed kernel extract (polyphenol as the biomarker) and lemongrass essential oil (citral as the biomarker).

Parameter	Time			
	0 month (Initial)	1 month	2 months	3 months
Appearance	Homogeneous, clear, transparent, and light yellowish color			
Polyphenol content	5560 μg (100%)	5521 ± 34 μg (99.3 ± 0.6%)	5508 ± 40 μg (99.1 ± 0.7%)	5493 ± 51 μg (98.8 ± 0.9%)
Citral amount	3.60 μg (100%)	3.47 ± 0.09 μg (96.4 ± 2.5%)	3.52 ± 0.08 μg (97.8 ± 2.2%)	3.44 ± 0.10 μg (95.6 ± 2.8%)

**Fig 4:**
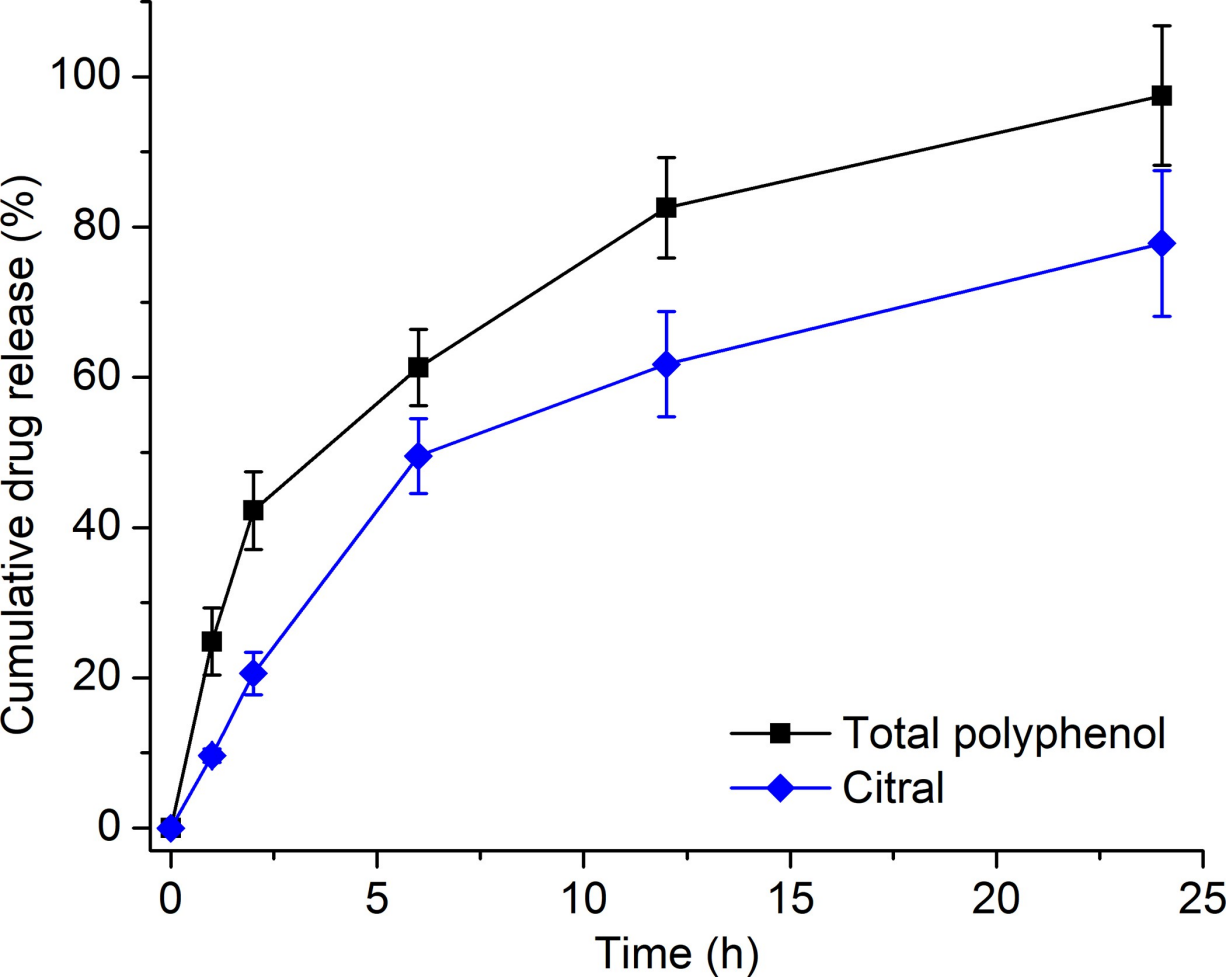
In-vitro Franz-cell cumulative drug release (%), in terms of the total polyphenol content (representing the mango seed extract) and citral (representing the lemongrass oil), of the microemulsion-based hydrogel (n = 3). The initial total amount of the polyphenol content and citral in the hydrogel was ~5560 μg and ~3.6 μg, respectively.

Regarding the in-vitro Franz-cell drug release test, the hydrogel demonstrated a sustained release pattern for both the polyphenol content and the citral amount (**Fig 4**). Within 12 h, the cumulative release percentages accounted for ~80% for polyphenol and ~60% for citral, and these amounts reached nearly 100% and 80%, respectively, at 24 h. This fact suggests that the microemulsion could both facilitate and control the drug release rates via the combined actions of both hydrophilic and lipophilic compositions [45]. The utilizations of both soybean oil and coconut oil in the formula could increase the system lipophilicity, thus, lipophilic compounds (i.e., citral) tend to stay in the microemulsion and their release rates were decelerated [44]. The prolonged release rates could be beneficial for the hydrogel applications as overnight anti-acne products.

### Irritation test

**Fig 5:**
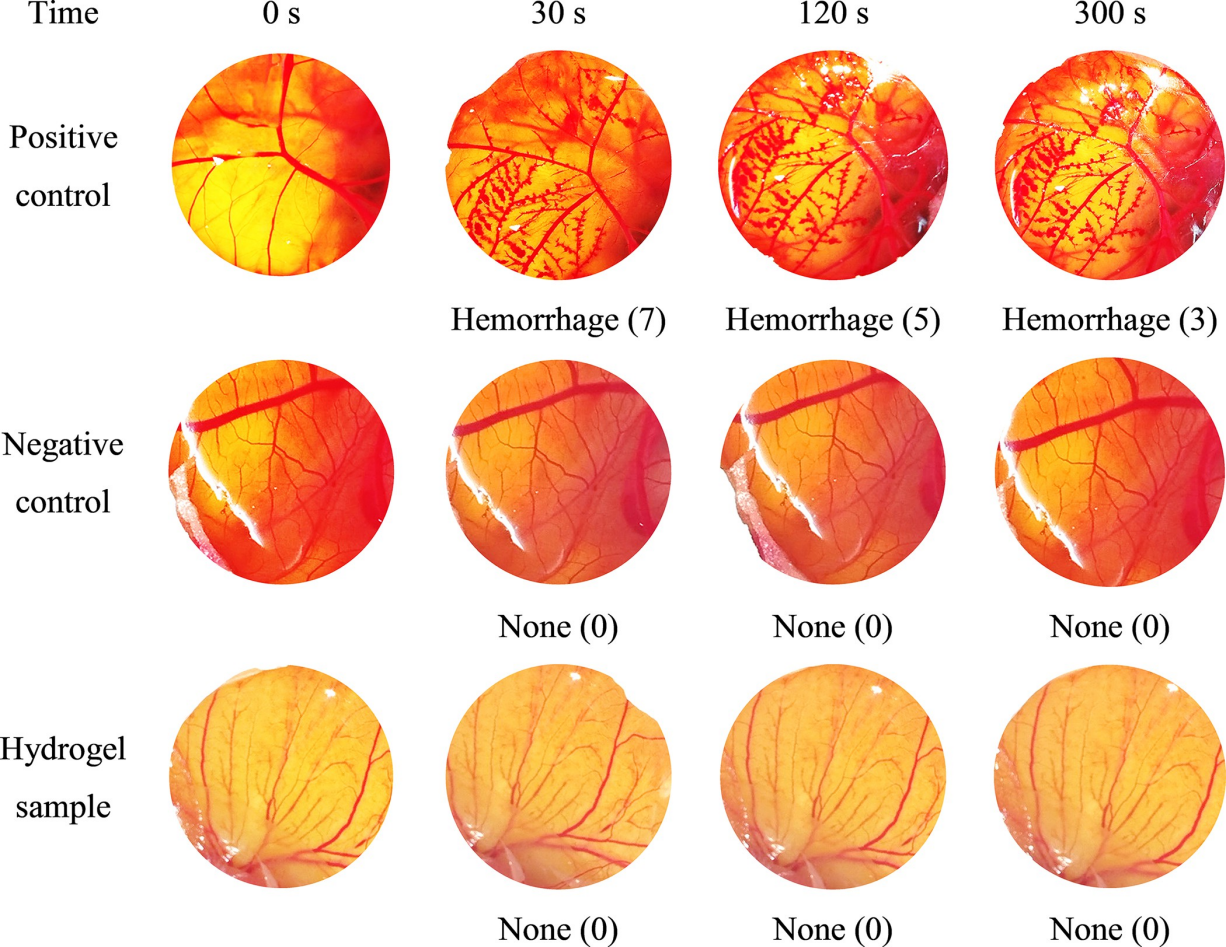
Photographs of HET-CAM test results, the positive control is sodium dodecyl sulfate 1% w/v (irritation score of 15), the negative control is NaCl 0.9% w/v (irritation score of 0), and the hydrogel sample is the microemulsion-based hydrogel containing mango seed kernel extract and lemongrass essential oil (irritation score of 0).

Safety is an important concern for topical drug delivery systems. The prohibition of employing animal testing in the cosmetics sector has created an immediate requirement for substitute testing techniques to evaluate novel products. The HET-CAM assay is a predictive model for in-vitro irritation test by observing the CAM vascular damage and calculating/evaluating the product irritation scores. HET-CAM has been confirmed as suitable for conducting eye irritation assessments due to its remarkable sensitivity [40]. Moreover, it can also be utilized to evaluate irritancy in various other mucous membranes, including the skin [55,56]. For this, **Fig 5** demonstrates that the negative control showed no irritation (score of 0), whereas the positive control showed severe irritation (score of 15 ± 0), suggesting the method reliability. Interestingly, the microemulsion-based hydrogel did not cause any observable vascular damage (score of 0). Since most of the marketed conventional topical products could cause skin irritation, especially when applying on the inflamed areas such as severe acne [33], our developed systems presented potential advantages of non-irritability, tolerability, and safeness for topical applications.

### In-vitro anti-inflammatory tests

To investigate the in-vitro anti-inflammatory actions of the microemulsion-based hydrogel, two assays were employed, the LPS-induced RAW 264.7 NO production inhibitory assay and the heat-induced BSA denaturation assay.

For the cell-based assay, the cytotoxic test (MTT test) was conducted to elucidate the safe concentration levels of the test samples (i.e., hydrogel) on the RAW 264.7 cells. To this end, both the hydrogel, the reference control L-NMMA, and the commercial product Klenzit-C^®^, were non-toxic to the cells at concentrations of up to 512 μg/mL, with the percentages of cell viability of 86.2 ± 2.5%, 96.8 ± 1.7%, and 92.1 ± 2.7%, respectively. According to the ISO 10993–5:2009 guideline, a sample possessing cell viability of > 80% is considered safe and could be used for biomedical purposes [57]. It is also worth to note that although the Cremophor RH40 is toxic to the RAW 264.7 cells, with an IC_50_ of 0.9788 mg/mL [58], its concentration in the hydrogel, at a hydrogel concentration of up to 512 μg/mL, was inadequate for causing cell damages. Hence, the samples were continued to be evaluated their NO production inhibitory action on LPS-induced RAW 264.7 cells.

**Fig 6:**
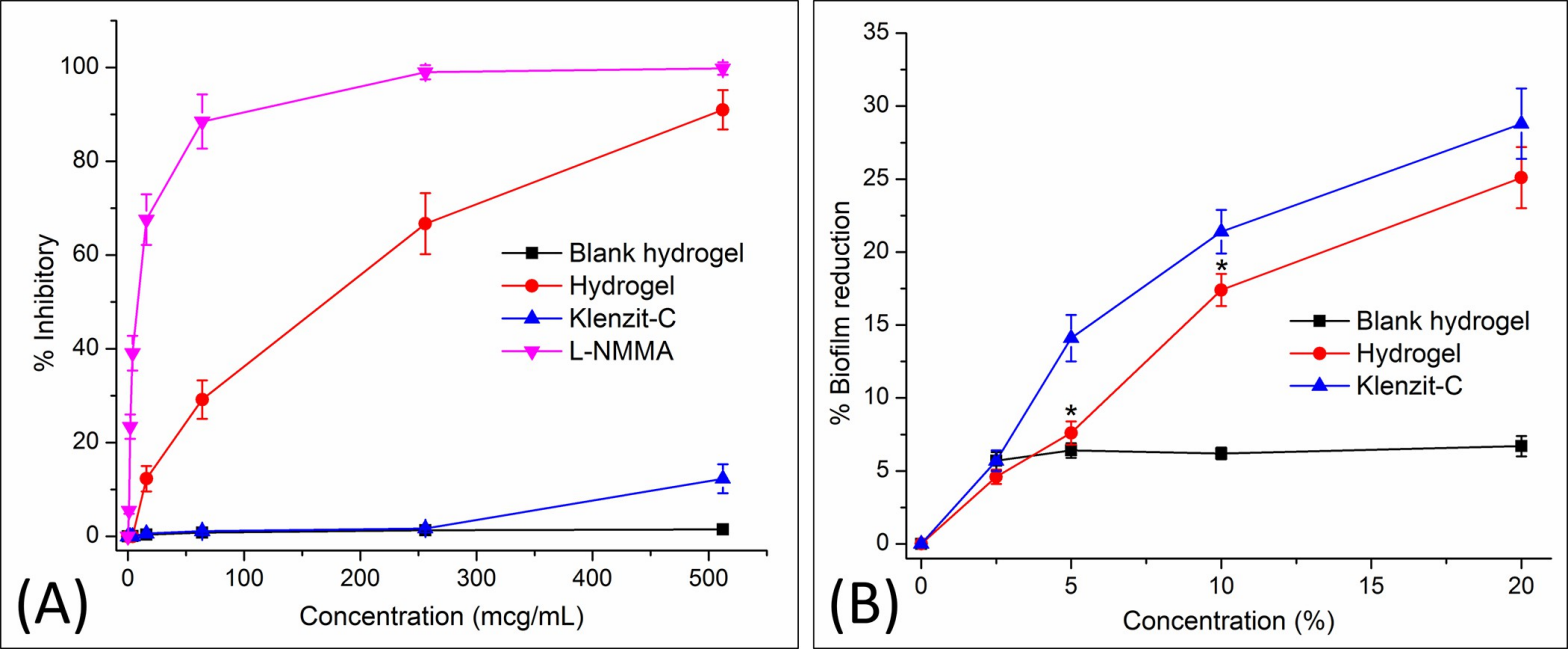
(A) LPS-induced RAW 264.7 cell NO production inhibitory percentages and (B) biofilm formation reduction percentages on *Cutibacterium acnes* of the blank hydrogel (hydrogel with no extract/oil), the hydrogel containing mango seed kernel extract and lemongrass essential oil, and the commercial product Klenzit-C^®^ (n = 3). * denotes significant different between the hydrogel and Klenzit-C^®^.

**Fig 6A** shows that the ability to inhibit NO production from LPS-induced RAW 264.7 cells follows the order of L-NMMA (reference control) > microemulsion-based hydrogel > Klenzit-C^®^ (reference commercial product) > blank hydrogel. Moreover, using the heat-induced BSA denaturation assay, a similar trend was observed, as diclofenac (reference control, IC_50_ = 6.64 ± 0.13 μg/mL) > microemulsion-based hydrogel (IC_50_ = 56.17 ± 0.44 μg/mL) > Klenzit-C^®^ (reference commercial product, IC_50_ = 270.85 ± 2.85 μg/mL) > blank hydrogel (IC_50_ > 512 μg/mL). Obviously, the blank hydrogel demonstrated no potential anti-inflammatory actions, indicating the therapeutic activity of the product was mainly generated from the bioactive compounds in the extracts. Additionally, the hydrogel possessed in-vitro anti-inflammatory actions in a concentration-dependent manner, according to the drug release patterns. Finally, compare to the commercial product, the hydrogel exhibited significantly greater anti-inflammatory effects. This could be advantageous for patients as it may help alleviate the redness, swelling, and discomfort associated with severe acne cases.

### In-vitro anti-biofilm formation test

Concerning the hydrogel antibacterial properties, the in-vitro anti-biofilm formation test was employed, with a comparison made to the commercial product Klenzit-C^®^ (**Fig 6B**). Firstly, the blank hydrogel showed little anti-biofilm effect (~5%), which might come from the bactericidal action of the lauric acid, the main component of the coconut oil, against *Cutibacterium acnes* [59]. Secondly, the hydrogel product and the commercial Klenzit-C^®^ possessed comparable concentration-dependent anti-biofilm actions, suggesting that the microemulsion-based hydrogel could preserve the bioactivities of the mango seed extract and the lemongrass oil, and these activities were adequately strong for the acne treatments. It is also worth to notice that citral, the main compounds in lemongrass essential oil, although possessing strong antibacterial activities, could induce skin irritation and cytotoxicity at concentrations of > 1% [16]. The present hydrogel had a citral amount of ~0.7%, thus, it was suitable for topical applications, with no potential irritability and cytotoxicity.

## Conclusions

This present work, for the first time, developed and in-vitro characterized microemulsion-based hydrogel containing mango seed kernel extract and lemongrass leaf essential oil as a topical pharmaceutical product for acne treatments. The optimal microemulsion could encapsulate 1% lemongrass oil and 10% mango extract, possessed a spherical droplet size of ~18.98 nm, and a zeta potential of -5.56 mV; and the final hydrogel product had a viscosity of ~3728 ± 237 cPs, a pH of 5.4-6.2, a spreadability of ~24 cm, an in-vitro Franz-cell controllable release rate of ~80% for polyphenol content and ~60% for citral within 12 h, and a good physicochemical stability of > 3 months. Biologically, the product showed no irritability, higher anti-inflammatory activities and similar anti-biofilm activity compared to the reference drug Klenzit-C^®^ (adapalene and clindamycin gel). In summary, the hydrogel showed much potentials to be a promising natural drug for the acne treatment

## Supporting information

S1 Fig The chromatogram and complete list of chemical constituents, analyzed by GC-MS/MS, of the lemongrass essential oil. (PDF)
