# Supplementary material for: Microemulsion-based topical hydrogels containing lemongrass leaf essential oil (Cymbopogon citratus (DC.) Stapf) and mango seed kernel extract (Mangifera indica Linn) for acne treatment: Preparation and in-vitro evaluations
Source: PLoS One. 2024 Oct 31;19(10):e0312841. doi: 10.1371/journal.pone.0312841 (PMC11527213; doi:10.1371/journal.pone.0312841)
Supplement: S1 Fig — (PDF) [file pone.0312841.s001.pdf]

Sample Information

Vial # : 1
Data File : D:\TN MOI TRUONG\KQTN\Nam 2023\Hoa Ly\Mau-0813.qgd

Chromatogram Unknown Sample D:\TN MOI TRUONG\KQTN\Nam 2023\Hoa Ly\Mau-0813.qgd

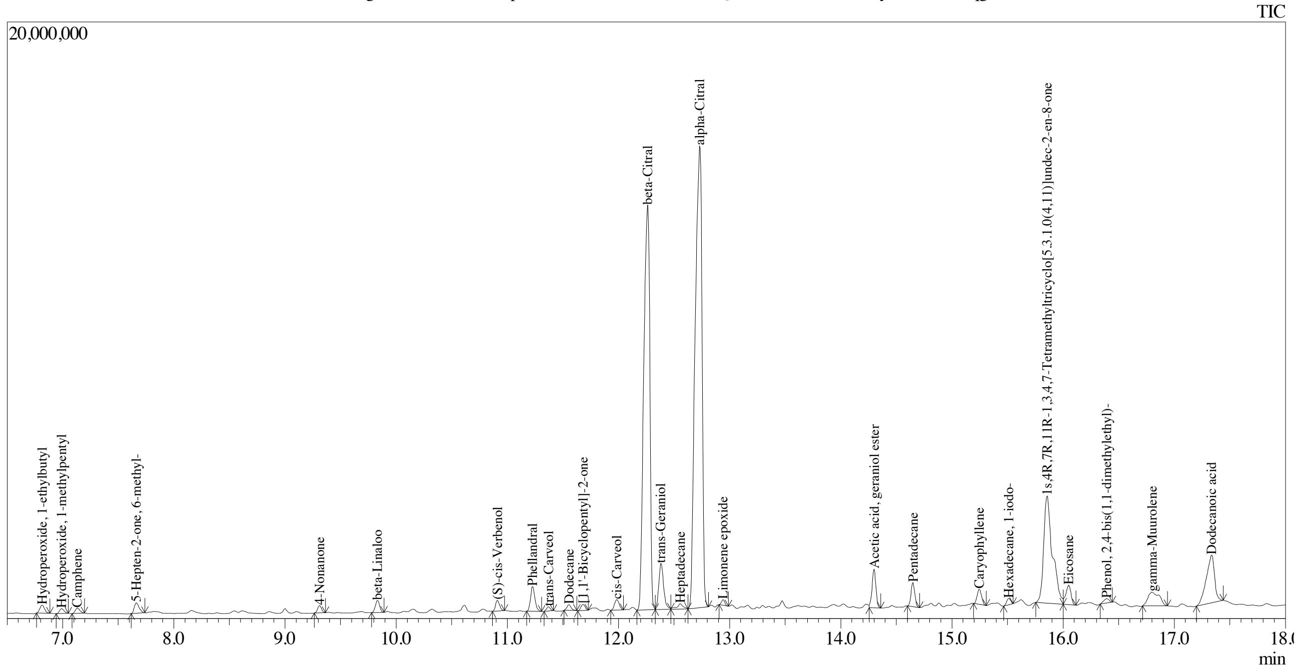

Peak Report TIC

Peak#	R.Time	Area	Area%	Height	Height%	Name
1	6.814	796263	0.46	265971	0.61	Hydroperoxide, 1-ethylbutyl
2	6.991	444095	0.26	162141	0.37	Hydroperoxide, 1-methylpentyl
3	7.132	491223	0.28	172892	0.40	Camphene
4	7.662	1081731	0.62	352116	0.81	5-Hepten-2-one, 6-methyl-
5	9.310	589961	0.34	238126	0.54	4-Nonanone
6	9.831	1064946	0.61	403948	0.92	beta-Linaloo
7	10.911	911802	0.52	361262	0.83	(S)-cis-Verbenol
8	11.227	2258309	1.30	823786	1.88	Phellandral
9	11.370	327513	0.19	136878	0.31	trans-Carveol
10	11.555	517960	0.30	196350	0.45	Dodecane
11	11.681	521278	0.30	202986	0.46	[1,1'-Bicyclopentyl]-2-one
12	11.985	1015852	0.58	352380	0.81	cis-Carveol
13	12.263	51699680	29.70	13509270	30.90	beta-Citral
14	12.383	4701915	2.70	1540838	3.52	trans-Geraniol
15	12.556	689701	0.40	177088	0.41	Heptadecane
16	12.732	66115967	37.98	15407071	35.24	alpha-Citral
17	12.941	382838	0.22	192619	0.44	Limonene epoxide
18	14.299	3092458	1.78	1291274	2.95	Acetic acid, geraniol ester
19	14.649	1881828	1.08	801684	1.83	Pentadecane
20	15.245	1382006	0.79	503228	1.15	Caryophyllene
21	15.512	524257	0.30	206663	0.47	Hexadecane, 1-iodo-
22	15.857	19468789	11.18	3579329	8.19	1s,4R,7R,11R-1,3,4,7-Tetramethyltricyclo[5.3.1.0(4,11)]undec-2-en-8-one
23	16.050	2098128	1.21	653920	1.50	Eicosane
24	16.392	500206	0.29	143491	0.33	Phenol, 2,4-bis(1,1-dimethylethyl)-
25	16.798	3227445	1.85	451685	1.03	gamma-Muurolene
26	17.337	8315164	4.78	1590869	3.64	Dodecanoic acid
		174101315	100.00	43717865	100.00	
